# Segment-specific prediction of vertebral collapse severity in osteoporotic fractures

**DOI:** 10.3389/fendo.2026.1867204

**Published:** 2026-08-07

**Authors:** Yitao Liao, Xingguo Zhang, Xuanyu Xie, Chao Li, Feng Qiu, Xian Zhang

**Affiliations:** 1 Department of Wuxi Affiliated Hospital, Nanjing University of Chinese Medicine, Nanjing, Jiangsu, China; 2 Department of Orthopedics, Wuxi Affiliated Hospital of Nanjing University of Chinese Medicine, Wuxi, China; 3 Department of Orthopedics, Xinwu District Traditional Chinese Medicine Hospital of Wuxi, Wuxi, China

**Keywords:** bone quality, muscle quality, osteoporotic vertebral compression fractures, segment-specific, vertebral collapse severity

## Abstract

**Background:**

Osteoporotic vertebral compression fractures (OVCFs) predominantly cluster at the thoracolumbar junction (T11-L1). Recent studies suggest that both bone quality and muscle health significantly contribute to vertebral collapse. However, among numerous multidimensional imaging metrics, which one can most accurately predict the severity of vertebral collapse, and whether the precise load-sharing proportions between bone and muscle exhibit distinct segment-specific differences, remain rarely quantified in the clinical literature.

**Purpose:**

To identify imaging metrics that best predict vertebral collapse severity and to quantify segment-specific contributions of bone and muscle quality at T11–L1.

**Methods:**

This retrospective cohort study analyzed 223 postmenopausal patients with single-level OVCFs at T11–L1. The severity of vertebral collapse was quantified by the sagittal area compression ratio of the fractured vertebral body (SACR-FVB). Radiological parameters including bone mineral density (BMD), trabecular CT Hounsfield units (CT Hu), vertebral bone quality (VBQ), paravertebral muscle quality (PVMQ), and fat infiltration rate (FIR) were measured. LASSO regression selected predictors; segment-specific multivariate regression and LMG variance decomposition quantified proportional contributions; RCS modeled dose-response relationships.

**Results:**

CT Hu and PVMQ were identified as significant independent predictors of SACR-FVB across all three evaluated segments (*P* < 0.05). LMG variance decomposition revealed that CT Hu consistently accounted for the largest proportion of the explained variance across all levels (68.2% at T11, 52.7% at T12, 56.3% at L1). However, the combined contribution of paraspinal muscle parameters (PVMQ and FIR) to the explained variance reached its maximum at the L1 segment (43.7%). Furthermore, RCS analyses confirmed that the structural vulnerability of the vertebral body manifests as a continuous gradient alongside local bone attenuation and progressive muscle deterioration.

**Conclusions:**

CT Hu and PVMQ were identified as significant independent predictors of vertebral collapse severity across all thoracolumbar junction segments. Localized bone quality, measured by CT Hu, remained the primary structural determinant, while the combined contribution of paraspinal muscle parameters increased caudally. Continuous quantitative monitoring of bone and muscle imaging metrics may enable early identification of high-risk patients before vertebral collapse occurs. These findings underscore the importance of a segment-specific, multidimensional approach to individualized fracture risk assessment and intervention.

## Introduction

Osteoporotic vertebral compression fractures (OVCFs) frequently induce intractable back pain and progressive kyphosis, and have become one of the important causes of disability and mortality among postmenopausal women worldwide (1, 2). Clinical observations consistently indicate that the distribution of OVCFs across the spine is not random, but highly concentrated in the T11-L1 vertebrae of the thoracolumbar junction (3, 4). As a key biomechanical fulcrum transitioning from the rigid thoracic cage to the highly mobile lumbar spine, this region bears complex axial loads and shear stresses (5). Once morphological collapse occurs in this region, it often leads to severe kyphosis, chronic back pain, and a significant decline in quality of life (6).

For a long time, the assessment of fracture risk has primarily relied on bone mineral density (BMD) measured by dual-energy X-ray absorptiometry (DXA). However, BMD cannot capture the three-dimensional trabecular microarchitecture and ignores the critical biomechanical contribution of paraspinal muscles, which act as an essential dynamic “tension band” to buffer axial compressive forces (7, 8). In recent years, high-resolution CT and magnetic resonance images (MRI) have enabled simultaneous, multidimensional evaluations of vertebral bone quality and muscle (9). Novel structural parameters, including trabecular CT Hounsfield unit (CT Hu), the vertebral bone quality (VBQ) score, paravertebral muscle quality (PVMQ), and the fat infiltration rate (FIR) of paraspinal muscles, have become superior independent predictors for assessing spinal instability (10–13).

Despite these advancements, current studies predominantly treat the thoracolumbar spine as a homogenous entity. Previous biomechanical and finite element analyses have shown that under axial loading, the internal stress distribution and the reliance on muscle tension vary significantly across different anatomical levels (14, 15). However, among numerous multidimensional imaging metrics, which one can most accurately predict the severity of vertebral collapse, and whether the precise load-sharing proportions between bone and muscle exhibit distinct segment-specific differences, remain rarely quantified in the clinical literature.

Therefore, the primary objective of this study was to quantitatively evaluate the segment-specific contributions of multidimensional bone and muscle parameters to the severity of vertebral collapse (the sagittal area compression ratio of the fractured vertebral body, SACR-FVB) at the thoracolumbar junction (T11-L1). This was achieved by combining exploratory penalized variable screening, segment-specific multivariable regression, and Lindeman, Merenda, and Gold (LMG) variance decomposition to quantify the relative contribution of bone- and muscle-related imaging parameters.

## Materials and methods

### Study design and ethical approval

This was a retrospective cross-sectional study approved by the Ethics Committee of Wuxi Hospital of Traditional Chinese Medicine. The study adhered to the Declaration of Helsinki and relevant ethical guidelines. A waiver of informed consent was granted by the hospital ethics committee.

### Patients

We reviewed the medical records of postmenopausal women aged 55–80 years who were hospitalized with OVCF between January 2016 and January 2026. Inclusion criteria were: 1. Complete clinical data. 2. Postmenopausal status (natural menopause). 3. Diagnosis of low bone mass or osteoporosis by DXA. 4. A single-level OVCF at T11–L1. 5. Fracture caused by low-energy trauma (Fall from standing height onto the buttocks). 6. No history of spinal surgery.

Exclusion criteria included: 1. High-energy trauma (e.g., traffic accidents or falls from significant height). 2. History of pelvic, hip, or spinal surgery. 3. Presence of a lumbosacral transitional vertebra. 4. Other spinal conditions (e.g., spinal tumors, spinal deformities, tuberculosis, or Kümmell disease). 5. Multiple vertebral fractures.

6. Neuromuscular disorders (e.g., dystrophy, ankylosing spondylitis, or Parkinson’s disease).

### Evaluation metrics

**Figure 1:**
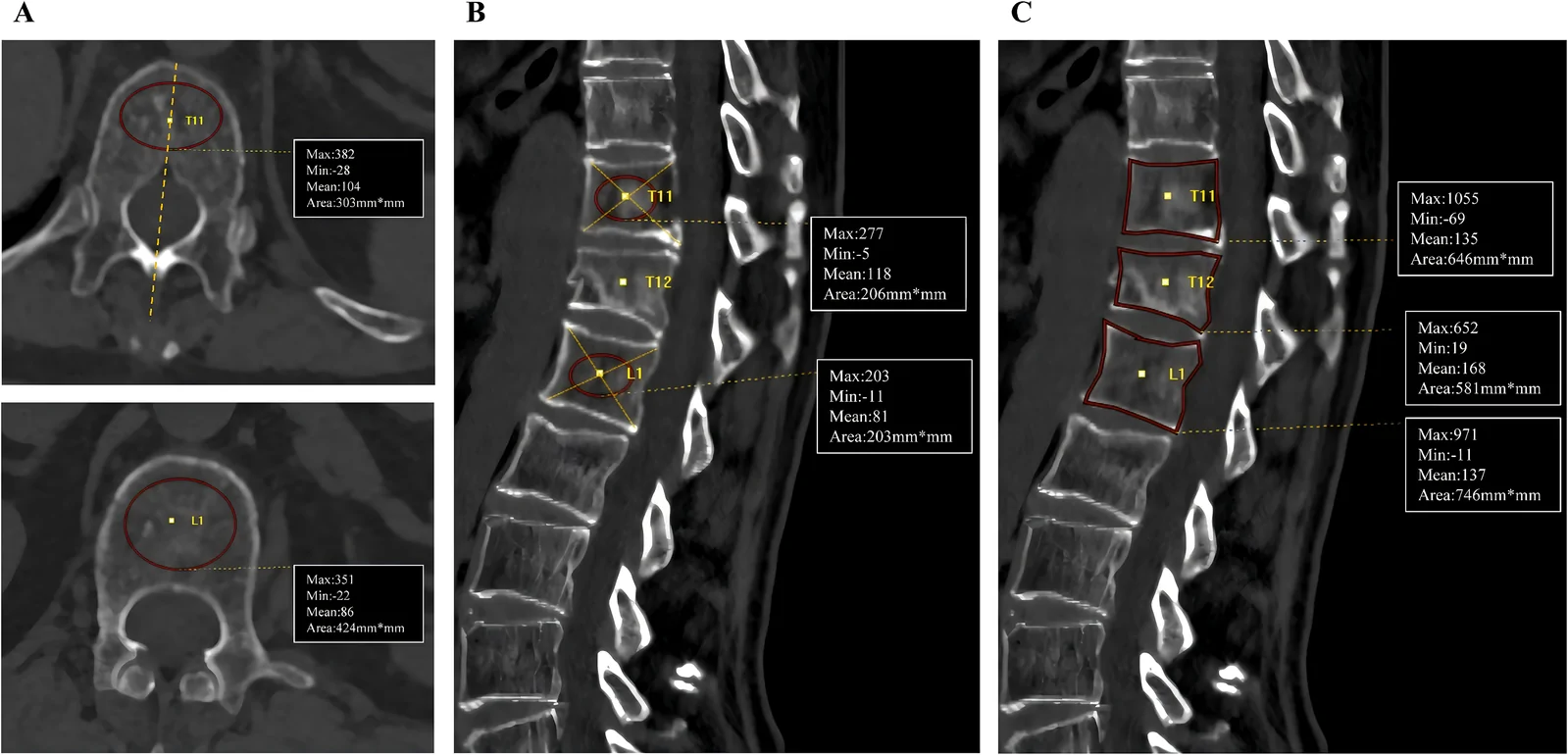
Measurement of the CT Hu value and SACR-FVB. **(A)** Axial CT Hu of the adjacent vertebral body. **(B)** Sagittal CT Hu of the adjacent vertebral body. **(C)** The FSCSA and the adjacent non-fractured vertebral bodies (NSCSA above and below). Fractured vertebral body CT Hu = (axial + sagittal CT Hu of superior vertebra + axial + sagittal CT Hu of inferior vertebra) / 4. The mean NSCSA was calculated as follows: NSCSA_mean_ = (NSCSA_above_ + NSCSA_below_) / 2. SACR-FVB was then calculated using the following formula: SACR-FVB = (NSCSA_mean_ – FSCSA) / NSCSA_mean_.

BMD was measured using DXA (L1-L4). Additionally, CT Hu values were obtained from both sagittal and axial CT images of the vertebrae adjacent to the fractured level (one above and one below). The average of these four measurements represented the CT Hu value of the fractured vertebra. The region of interest (ROI) was defined using an elliptical shape placed at the intersection of two diagonals across the vertebral body (**Figures 1A, 1B**).

We measured the sagittal cross-sectional area of the fractured vertebral body (FSCSA) and the adjacent non-fractured vertebral bodies (NSCSA above and below). The average of the NSCSA values was calculated as:


$${\text{NSCSA}}_{\text{mean}}=\;\left({\text{NSCSA}}_{\text{above}}+{\text{ NSCSA}}_{\text{below}}\right)/2$$

The sagittal area compression ratio of the fractured vertebral body (SACR-FVB) was then defined as:


$$\text{SACR}-\text{FVB}=\left({\text{NSCSA}}_{\text{mean}}–\text{ FSCSA}\right)/{\text{NSCSA}}_{\text{mean}}$$

This metric quantifies the relative degree of vertebral body collapse (**Figure 1C**).

**Figure 2:**
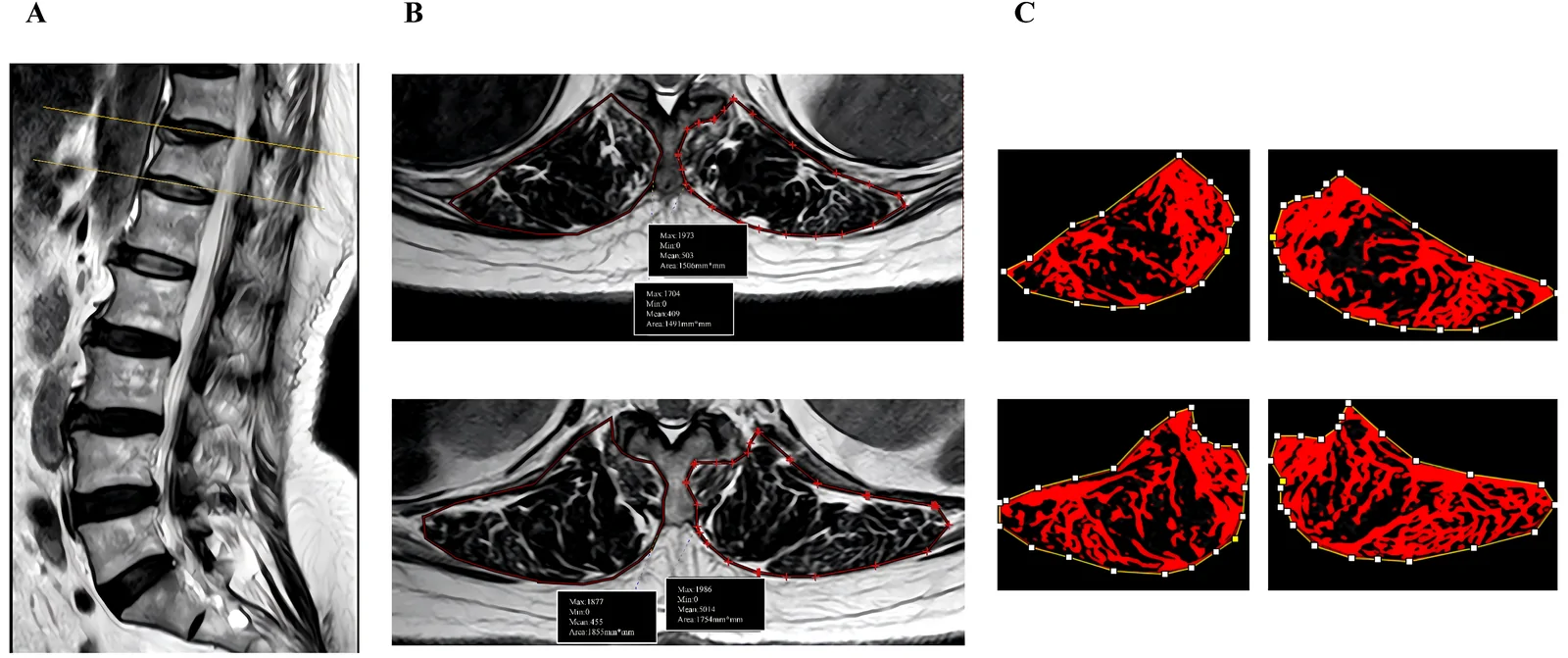
Measurement of the FIR of the paraspinal muscles. **(A)** Level at which muscle parameters were measured. **(B)** ROI for measurement. **(C)** FIR measured using Image J. The boundaries of the bilateral erector spinae and multifidus muscles were manually delineated, and the FIR was calculated using Image J software. FIR_mean_ = (FIR_above_ + FIR_below_)/2.


We assessed the FIR of paraspinal muscles at levels adjacent to the fracture (above and below) using previously validated methods, The average FIR was calculated as (**Figure 2**):

$${\text{FIR}}_{\text{mean}}=\left({\text{FIR}}_{\text{above}}+{\text{ FIR}}_{\text{below}}\right)/2$$

The total cross-sectional area of paraspinal muscles (TCSAPM) was measured by manually delineating a ROI along the fascial boundaries of the bilateral multifidus and erector spinae muscles. The functional cross-sectional area of paraspinal muscles (FCSAPM), representing the lean muscle mass, was subsequently calculated using the following formula: FCSAPM = TCSAPM× (1 - FIR).

**Figure 3:**
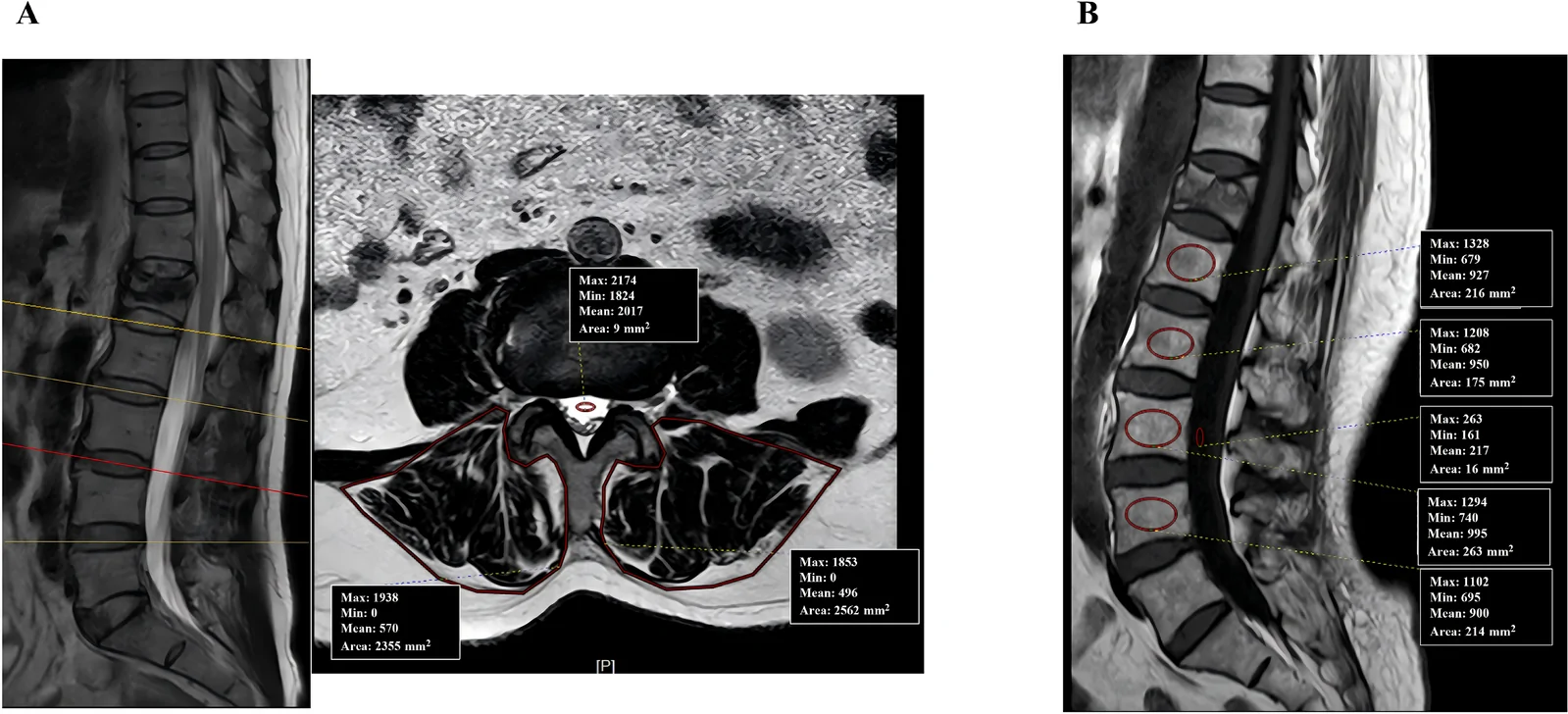
MRI-based measurement of VBQ and PVMQ scores. **(A)** Representative axial T2-weighted image illustrating the PVMQ measurement. ROIs are manually delineated along the anatomical boundaries of the bilateral erector spinae and multifidus muscles. A reference ROI is positioned within the CSF at the corresponding level to extract the baseline SI. PVMQ = SI_L1/2-L4/5_/SI_L3/4CSF_. **(B)** Representative median sagittal T1-weighted image illustrating the VBQ measurement. ROIs are placed within the cancellous bone of L1 to L4 vertebrae and the CSF at the L3 level to obtain the mean SI. VBQ = SI_L1-L4_/SI_L3CSF_.

PVMQ was evaluated using axial T2-weighted MRI (12). Intermediate cross-sectional images at four intervertebral disc levels, from L1/L2 to L4/L5, were selected for analysis. ROIs were manually delineated along the boundaries of the right and left erector spinae and multifidus muscles. The signal intensity (SI) values of the bilateral muscles were averaged to obtain the measurement for each individual disc level. The SI of the cerebrospinal fluid (CSF) at the L3/L4 transect level was additionally measured to serve as an adjusted indicator of baseline signal differences. The final PVMQ score was calculated by dividing the overall mean SI of the paravertebral muscles across the four segments (L1/L2 to L4/L5) by the SI of the L3 CSF, according to the following formula: PVMQ = SI_L1/2-L4/5_/SI_L3/4CSF_ (**Figure 3A**).

VBQ was evaluated using median sagittal T1-weighted MRI of the lumbar spine (16). ROIs were placed within the cancellous bone region of the L1 to L4 vertebrae to calculate the mean SI. Focal lesions and the posterior venous plexus were strictly excluded. An additional ROI was positioned within the CSF at the L3 level to obtain a reference SI. The VBQ score was calculated by dividing the average SI of the L1-L4 vertebrae by the SI of the L3 CSF, according to the following formula: VBQ = SI_L1-L4_/SI_L3CSF_ (**Figure 3B**).

Two independent examiners performed the measurements. To assess inter-observer consistency, intraclass correlation coefficients (ICCs) were calculated using a two-way mixed-effects model.

### Statistical analyses

Continuous variables are expressed as the mean and standard deviation. Pearson correlation analysis was performed to evaluate the associations between imaging parameters and SACR-FVB. LASSO regression was applied independently to each vertebral segment (T11, T12, and L1) as an exploratory penalized screening method. A 10-fold cross-validation procedure was used to determine the optimal tuning parameter (λ). Variables with non-zero coefficients at the optimal λ value were retained for subsequent exploratory multivariable regression. Segment-specific multivariable linear regression models were constructed using the variables retained by LASSO, with SACR-FVB as the dependent variable. Relative importance analysis was performed using the Lindeman, Merenda, and Gold (LMG) method to calculate the proportional contribution of each retained variable to the explained variance. To assess robustness without data-driven variable selection, conventional forced-entry multivariable regression was additionally performed for each segment using clinically predefined predictors, including age, BMI, BMD, CT Hu, VBQ, PVMQ, and FIR. Variance inflation factors were calculated to assess multicollinearity, and LMG analysis was repeated in these models. Restricted cubic spline (RCS) models with three knots were used to evaluate dose-response relationships between CT Hu, PVMQ, and SACR-FVB. ANOVA was used to test for non-linearity. Exploratory segment-specific reference values were further derived from the fitted RCS curves by identifying the approximate CT Hu or PVMQ values corresponding to a predicted SACR-FVB of 20%, with other covariates fixed at their median values. Statistical analyses and visualizations were performed using RStudio 2025.05.0 + 496 with the glmnet, relaimpo, rms, and ggplot2 packages. Descriptive analyses were performed using SPSS Statistics version 25.0. All statistical tests were two-sided, and P < 0.05 was considered statistically significant.

## Results

### Patient characteristics

A total of 223 patients meeting the inclusion and exclusion criteria were ultimately enrolled in the study. Among them, 85 patients presented with T11 vertebral compression fractures, with a mean age of 70.61 ± 4.99 years and a mean body mass index (BMI) of 23.49 ± 2.11 kg/m². Seventy-two patients had T12 fractures, with a mean age of 69.21 ± 4.92 years and a mean BMI of 23.89 ± 2.10 kg/m². The L1 fracture group consisted of 66 patients, with a mean age of 70.48 ± 2.85 years and a mean BMI of 23.96 ± 1.72 kg/m². Detailed demographic and baseline clinical characteristics of the patient population are summarized in **Table 1**.

**Table 1:** Patient demographics.

Characteristic	T11(n=85)		T12(n=72)		L1(n=66)	
	Mean	SD	Mean	SD	Mean	SD
AGE (years)	70.61	4.99	69.21	4.92	70.48	2.85
Age at Menopause(years)	53.11	3.06	52.65	3.08	53.44	3.02
Postmenopausal duration (years)	17.51	5.96	16.56	5.69	17.05	4.18
BMI (kg/m^2^)	23.49	2.11	23.89	2.10	23.96	1.72
SACR-FVB (%)	21.65	4.86	20.93	4.55	23.71	6.61
BMD (T)	-3.59	0.43	-3.4	0.42	-3.35	0.45
CT Hu	91.25	13.00	94.96	12.78	99.50	15.52
VBQ score	3.464	0.607	3.363	0.796	3.874	0.673
TCSAPM (mm^2^)	1537.39	188.03	1505.40	294.25	1495.24	312.24
FCSAPM (mm^2^)	925.87	130.99	1018.11	290.30	982.06	213.85
FIR (%)	39.71	6.08	32.31	7.86	33.99	7.67
PVMQ score	0.380	0.059	0.327	0.076	0.332	0.080
TP1NP (ng/mL)	48.54	15.33	47.67	14.90	48.77	14.40
OCN (ng/mL)	28.11	10.78	28.08	10.87	28.11	10.56
βCROSSL (ng/mL)	0.528	0.213	0.516	0.225	0.518	0.224
PTH (pg/mL)	34.11	13.03	36.07	13.89	36.01	15.33
25-OH VitD (nmol/mL)	19.19	5.63	19.21	5.13	19.804	5.43

BMI, Body mass index; SACR-FVB, The sagittal area compression ratio of the fractured vertebral body; BMD, Bone mineral density; CT Hu, CT Hounsfield unit; VBQ, Vertebral bone quality; TCSAPM, Total cross-sectional area of paraspinal muscles; FCSAPM, Functional cross-sectional area of paraspinal muscles; FIR, Fatty infiltration ratio; PVMQ, Paravertebral muscle quality; TP1NP, Total procollagen type I N-terminal propeptide; OCN, Osteocalcin; CROSSL, C-terminal telopeptide of type I collagen; PTH, Parathyroid hormone; 25-OH VitD, 25-Hydroxy vitamin D.

Based on the ICC analysis, inter-rater reliability for the measurements of CT Hu, SACR-FVB, FIR, PVMQ, and VBQ across the T11, T12, and L1 vertebral segments was generally good to excellent. For the T11 segment, ICC values for CT Hu, SACR-FVB, FIR, PVMQ, and VBQ were 0.867, 0.883, 0.802, 0.897 and 0.913 respectively. For the T12 segment, ICC values for CT Hu, SACR-FVB, FIR, PVMQ, and VBQ were 0.841, 0.804, 0.773, 0.867 and 0.874 respectively. For the L1 segment, ICC values were 0.834 for CT Hu, 0.805 for SACR-FVB, 0.827 for FIR, 0.873 for PVMQ, and 0.885 for VBQ.

### Correlation analysis and exploratory LASSO screening

**Figure 4:**
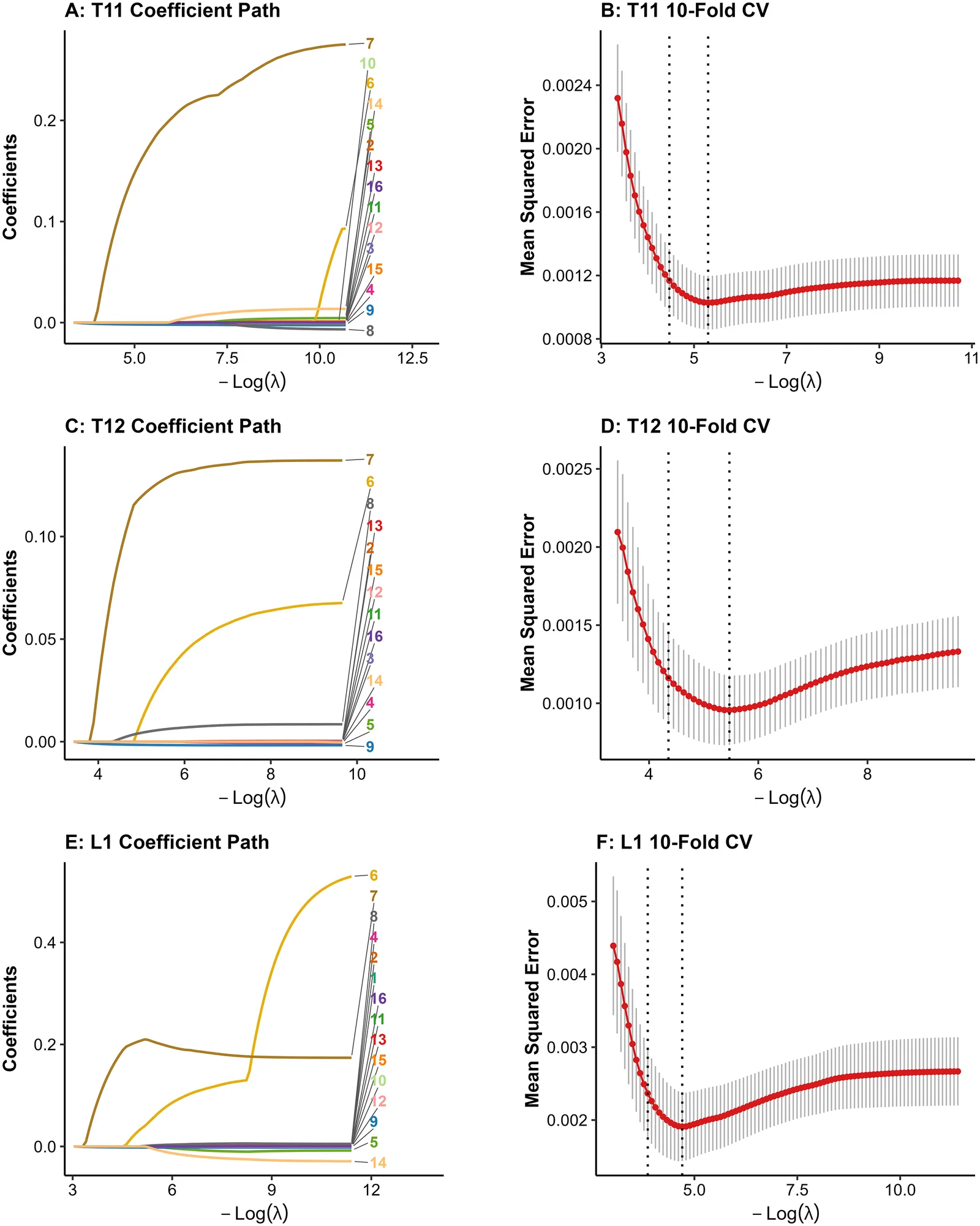
Feature selection via LASSO regression across T11, T12, and L1 segments. **(A, C, E)** LASSO coefficient path plots for the T11, T12, and L1 segments, respectively. Each curve depicts the coefficient trajectory of an individual variable across varying penalization parameters (-Log(λ)). **(B, D, F)** Ten-fold cross-validation curves for the corresponding segments. The MSE is plotted against -Log(λ). Solid red dots indicate the average MSE, with vertical error bars representing the standard deviation. Vertical dashed lines denote the optimal λ values selected by the cross-validation models. 1 = Age, 2 = age at menopause, 3 = postmenopausal duration, 4 = BMI, 5 = BMD, 6 = FIR, 7 = PVMQ, 8 = VBQ, 9 = CT Hu, 10 = TCSAPM, 11 = FCSAPM, 12 = TP1NP, 13 = OCN, 14 = β-CROSSL, 15 = PTH, and 16 = 25-OH VitD.

Pearson correlation analysis revealed the bivariate associations between clinical and radiological parameters and SACR-FVB across the T11, T12, and L1 segments (**Table 2**). Across all three segments, CT Hu and BMD demonstrated significant negative correlations with SACR-FVB, whereas VBQ, PVMQ, and FIR exhibited significant positive correlations (*P* < 0.05). The FCSAPM demonstrated a significant negative correlation with SACR-FVB exclusively in the T11 segment (*r* = -0.309, *P* = 0.004). Bone turnover markers, age, BMI, and other demographic variables did not show significant linear correlations with SACR-FVB. The LASSO regression was executed independently for each segment as an exploratory penalized screening procedure to derive parsimonious candidate models from a moderately sized and potentially correlated imaging variable set. Variables yielding non-zero coefficients at the optimal penalization parameter, determined via 10-fold cross-validation, were retained for subsequent exploratory multivariable modeling (**Figure 4**). The LASSO procedure retained CT Hu, PVMQ, and TCSAPM for the T11 segment; CT Hu, VBQ, PVMQ, and FIR for the T12 segment; and CT Hu, PVMQ, and FIR for the L1 segment (**Table 2**).

**Table 2:** Pearson correlation analysis between SACR-FVB and each factor.

Variable	T11(n=85)			T12(n=72)			L1(n=66)		
	*r*	*P-*value	LASSO result	*r*	*P-*value	LASSO result	*r*	*P-*value	LASSO result
Age	0.153	0.161	-	-0.123	0.302	-	-0.122	0.327	-
Age at menopause	-0.012	0.916	-	0.042	0.728	-	0.080	0.524	-
Postmenopausal duration	0.134	0.220	-	-0.129	0.279	-	-0.143	0.254	-
BMI	-0.193	0.077	-	-0.221	0.063	-	-0.041	0.074	-
BMD	**-0.368**	**0.001**	-	**-0.418**	**<0.001**	-	**-0.433**	**<0.001**	**-**
CT Hu	**-0.733**	**<0.001**	**Selected**	**-0.720**	**<0.001**	**Selected**	**-0.738**	**<0.001**	**Selected**
VBQ	**0.556**	**<0.001**	-	**0.535**	**<0.001**	**Selected**	**0.316**	**0.010**	**-**
PVMQ	**0.554**	**<0.001**	**Selected**	**0.589**	**<0.001**	**Selected**	**0.645**	**<0.001**	**Selected**
FIR	**0.483**	**<0.001**	**-**	**0.413**	**<0.001**	**Selected**	**0.414**	**0.001**	**Selected**
TCSAPM	0.039	0.726	**Selected**	-0.025	0.835	-	0.026	0.837	-
FCSAPM	**-0.309**	**0.004**	-	-0.186	0.117	-	-0.213	0.087	-
TP1NP	0.078	0.481	-	0.065	0.587	-	-0.139	0.265	-
OCN	0.060	0.583	-	-0.036	0.766	-	0.042	0.737	-
βCROSSL	-0.016	0.884	-	-0.059	0.625	-	-0.174	0.162	-
PTH	-0.094	0.390	-	0.037	0.757	-	-0.052	0.677	-
25-OH VitD	0.187	0.086	-	-0.074	0.537	-	0.059	0.639	-

“-” mean Excluded. Significant values are highlighted in bold.

### Segment-specific multivariate linear regression models

Exploratory multivariable linear regression models were constructed for the T11, T12, and L1 segments using the variables retained by LASSO to estimate their associations with SACR-FVB (**Tables 3**–**5**). All three segment-specific models demonstrated overall statistical significance (*P* < 0.001). In the T11 segment model (*R^2^* = 0.614, Adjusted *R^2^* = 0.599), CT Hu (*β* = -0.615, *P* < 0.001) and PVMQ (*β* = 0.285, *P* = 0.007) showed significant associations with SACR-FVB, whereas TCSAPM did not reach statistical significance (*P* = 0.076). In the T12 segment model (*R^2^* = 0.626, Adjusted *R^2^* = 0.604), CT Hu (*β* = -0.506, *P* < 0.001) and PVMQ (*β* = 0.235, *P* = 0.026) showed significant associations with SACR-FVB. VBQ and FIR were retained by the exploratory LASSO procedure but did not show statistical significance in the subsequent multivariable model (*P* = 0.108 and *P* = 0.259, respectively). In the L1 segment model (*R^2^* = 0.629, Adjusted *R^2^* = 0.612), CT Hu (*β* = -0.551, *P* < 0.001) and PVMQ (*β* = 0.279, *P* = 0.011) showed significant associations with SACR-FVB, while FIR, although retained by the exploratory LASSO procedure, was not statistically significant (*P* = 0.260). Across all three evaluated segments, CT Hu consistently showed a significant negative association with SACR-FVB, whereas PVMQ consistently showed a significant positive association.

**Table 3:** Multiple linear regression analysis for predictors of SACR-FVB at T11 level.

Variable	*R*^2^ = 0.614, Adjusted *R*^2^ = 0.599, *p* value <0.001					Correlation	
	B	SE B	*β*	*t*	*p-*value	Partial	Part
CT Hu	-0.002	0.000	-0.615	-7.919	**<0.001**	-0.661	-0.547
PVMQ	0.233	0.064	0.285	3.670	**0.007**	0.378	0.253
TCSAPM	0.000	0.000	0.125	1.800	0.076	0.196	0.124

B, unstandardized regression coefficients; SE, standard error; β standardized regression coefficients; Significant values are highlighted in bold.

**Table 4:** Multiple linear regression analysis for predictors of SACR-FVB at T12 level.

Variable	*R*^2^ = 0.626, Adjusted *R*^2^ = 0.604, *p* value <0.001					Correlation	
	B	SE B	*β*	*t*	*p-*value	Partial	Part
CT Hu	-0.002	0.000	-0.506	-5.336	**<0.001**	-0.546	-0.399
PVMQ	0.140	0.061	0.235	2.277	**0.026**	0.268	0.170
VBQ	0.008	0.005	0.147	1.630	0.108	0.195	0.122
FIR	0.063	0.055	0.108	1.138	0.259	0.138	0.085

B, unstandardized regression coefficients; SE, standard error; β, standardized regression coefficients; Significant values are highlighted in bold.

**Table 5:** Multiple linear regression analysis for predictors of SACR-FVB at L1 level.

Variable	*R*^2^ = 0.629, Adjusted *R*^2^ = 0.612, *p* value <0.001					Correlation	
	B	SE B	*β*	*t*	*p-*value	Partial	Part
CT Hu	-0.002	0.000	-0.551	-5.890	**<0.001**	-0.599	-0.455
PVMQ	0.229	0.087	0.279	2.619	**0.011**	0.316	0.202
FIR	0.090	0.079	0.105	1.138	0.260	0.143	0.088

B, unstandardized regression coefficients; SE, standard error; β, standardized regression coefficients; Significant values are highlighted in bold.

Sensitivity analyses using conventional forced-entry multivariable regression models yielded broadly consistent findings. In these models, age, BMI, BMD, CT Hu, VBQ, PVMQ, and FIR were simultaneously entered as clinically predefined predictors. CT Hu remained a significant negative predictor of SACR-FVB across T11, T12, and L1, whereas PVMQ remained a significant positive predictor across all three segments. The adjusted R^²^ values of the forced-entry models were 0.571, 0.589, and 0.596 for T11, T12, and L1, respectively. No substantial multicollinearity was observed, with all variance inflation factors below 3. Repeated LMG analysis further showed that CT Hu remained the largest contributor to the explained variance across all three segments. Detailed results are provided in **Supplementary Tables 1**-**3**.

### Relative importance of independent predictors

**Figure 5:**
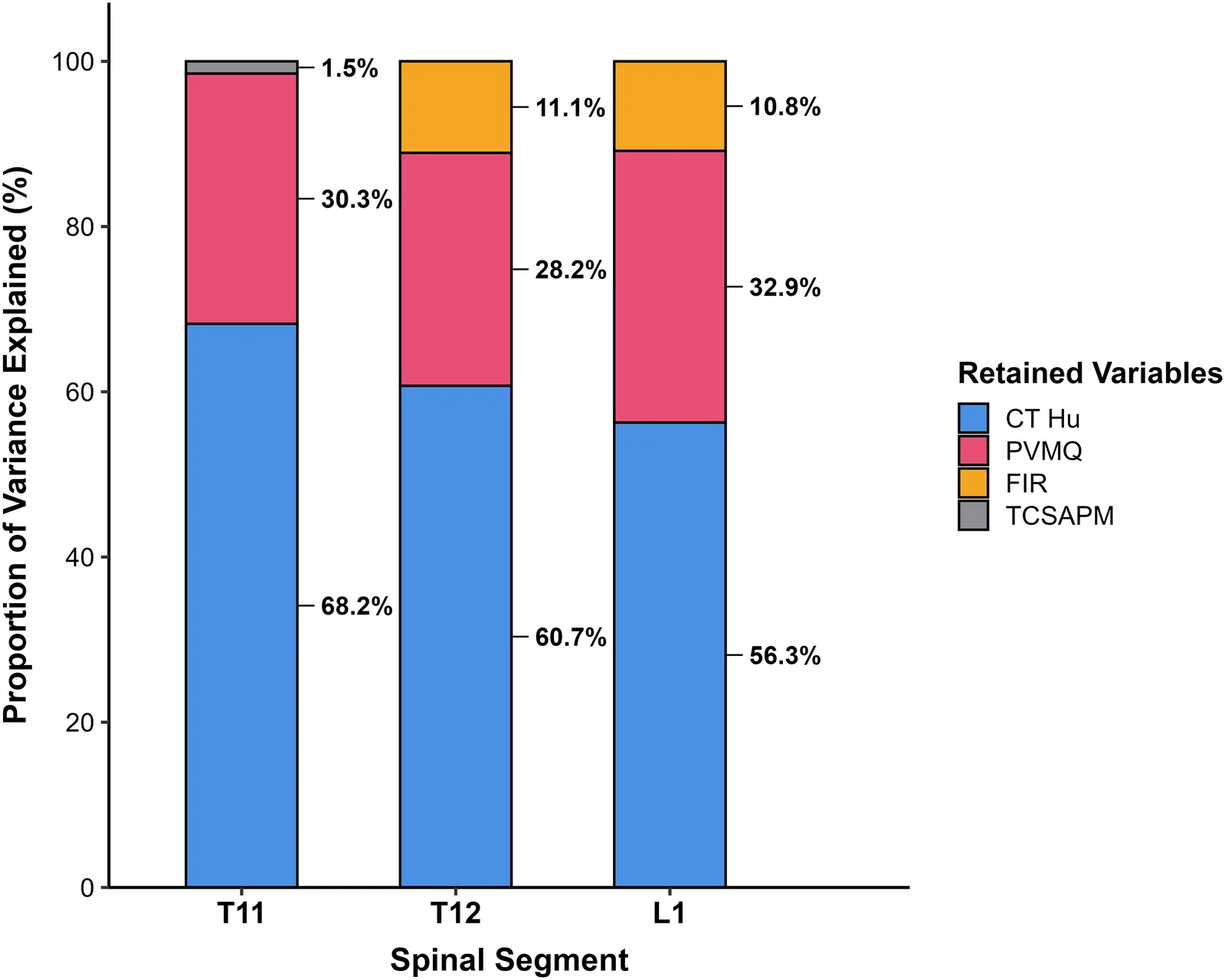
Proportional contribution of independent predictors to the SACR-FVB across T11-L1 spinal segments. A stacked bar chart displays the relative variance in SACR-FVB explained by local bone and muscle parameters at the T11, T12, and L1 levels. Values within the bars denote the normalized relative importance of each predictor, expressed as a percentage of the total explained variance.

To quantify the proportional contribution of each independent predictor to the total explained variance of SACR-FVB, the LMG relative importance analysis was performed for each segment-specific multivariate model (**Figure 5**). In the T11 segment, CT Hu accounted for the largest proportion of the explained variance (68.2%), followed by PVMQ (30.3%) and TCSAPM (1.5%). In the T12 segment, CT Hu remained the primary contributor, accounting for 52.7% of the variance. The remaining variance in the T12 model was distributed among PVMQ (21.5%), VBQ (17.4%), and FIR (8.3%). For the L1 segment, the total explained variance was apportioned among CT Hu (56.3%), PVMQ (32.9%), and FIR (10.8%). Across the evaluated segments, CT Hu and PVMQ consistently provided the largest proportional contributions to the models.

### Continuous dose-response relationships evaluated by RCS

**Figure 6:**
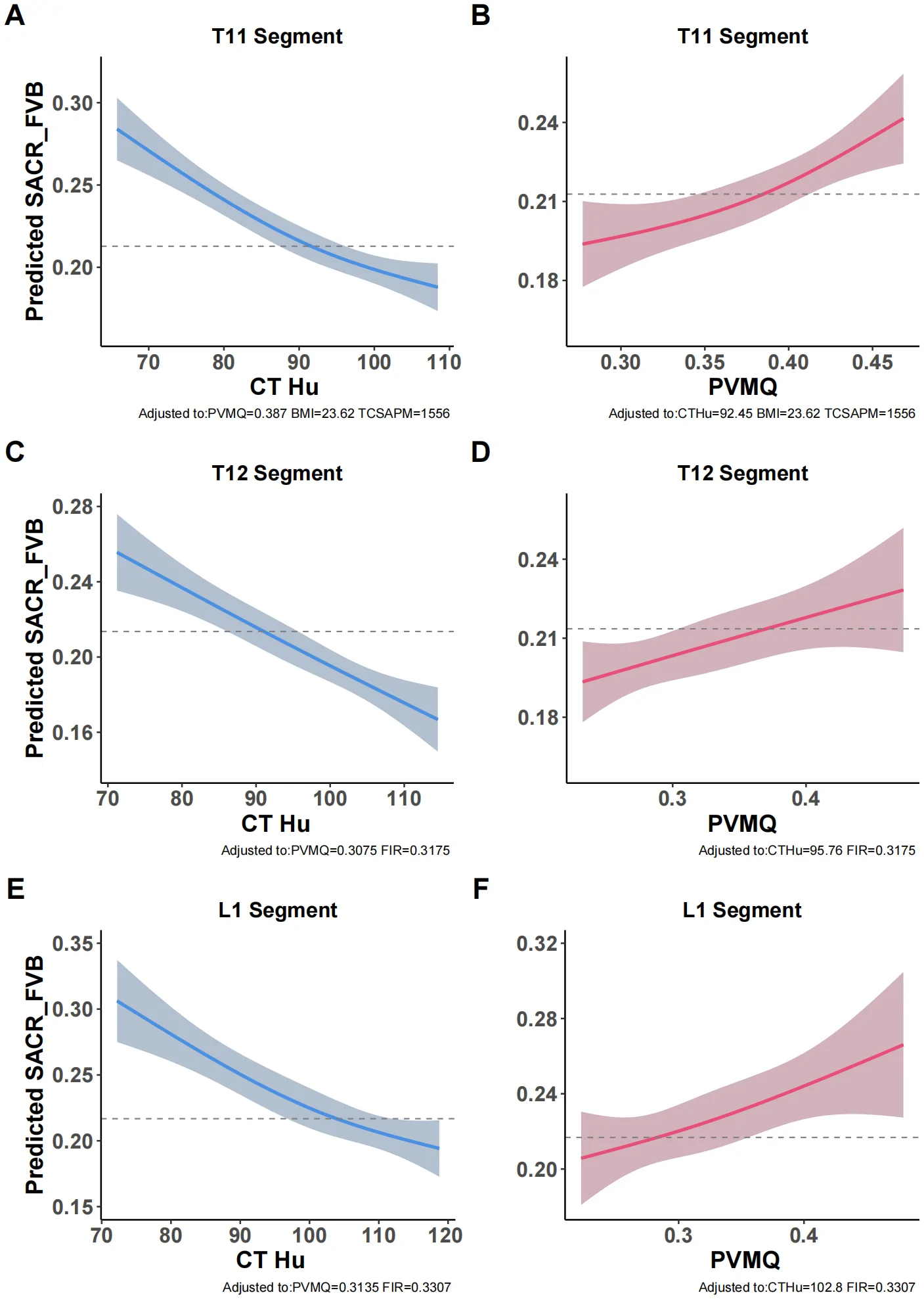
RCS curves illustrating the dose-response relationships between core predictors and the SACR-FVB across T11-L1 segments. **(A, C, E)** Dose-response curves representing the association between CT Hu and predicted SACR-FVB at the T11, T12, and L1 levels, respectively. **(B, D, F)** Dose-response curves representing the association between PVMQ and predicted SACR-FVB at the corresponding segmental levels. Solid lines indicate the predicted values, and shaded bands represent the 95% confidence intervals. Dashed horizontal lines indicate the median SACR-FVB reference values. All models were adjusted for segment-specific concurrent covariates, as specified below each respective x-axis.

RCS analyses showed continuous and monotonic dose-response relationships between the core imaging parameters and SACR-FVB across the three evaluated segments (**Figures 6A-F**). ANOVA tests for non-linearity did not identify statistically significant non-linear deviations. The P values for non-linearity for CT Hu were 0.086, 0.872, and 0.270 at T11, T12, and L1, respectively. The corresponding P values for PVMQ were 0.292, 0.983, and 0.774, respectively, indicating no significant non-linear inflection points. To improve clinical interpretability, we further derived exploratory segment-specific reference values from the fitted RCS models by identifying the approximate predictor values corresponding to a predicted SACR-FVB of 20%, with other covariates fixed at their median values. The 20% level was selected because the Genant semi-quantitative grading system defines mild vertebral fracture as approximately 20%–25% vertebral height loss (17). The exploratory reference values for CT Hu were 99.1 HU, 97.7 HU, and 114.4 HU at T11, T12, and L1, respectively. The corresponding values for PVMQ were 0.323, 0.277, and 0.201, respectively (**Supplementary Table 4**). These values should be interpreted as exploratory risk-reference values rather than validated clinical thresholds.

## Discussion

The concurrent structural degeneration of vertebral bone and paraspinal musculature significantly exacerbates the risk and severity of OVCF (18). Recent biomechanical and radiological advancements have shifted the diagnostic focus from isolated BMD assessments to a multidimensional, comprehensive evaluation of bone and muscle quality (19). Evidence indicates that the paraspinal musculature not only maintains dynamic sagittal balance but also imparts a critical mechanical tension on the spine, thereby buffering unconstrained axial compression and mitigating fracture progression (20). Although independent structural parameters, such as trabecular CT Hu, the VBQ score, and intramuscular FIR, have been individually linked to spinal instability, vertebral compression fractures predominantly occur at the thoracolumbar junction (3, 21–23). In fact, previous biomechanical and finite element studies have demonstrated that the internal stress distribution and the reliance on paraspinal muscular tension vary significantly across different anatomical levels of the thoracolumbar spine when subjected to complex axial and shear loads (24, 25). However, among various radiological bone and muscle metrics, which specific parameters most accurately predict the severity of vertebral collapse, and what the precise load-sharing proportions are between bone and muscle at specific anatomical segments, have rarely been investigated.

This study quantitatively evaluated the segment-specific contributions of bone and muscle parameters to the degree of SACR-FVB in the thoracolumbar spine (T11-L1). To comprehensively capture these structural properties, CT Hu and the VBQ score were utilized as indicators of bone quality, while PVMQ, FIR, and cross-sectional areas (TCSAPM, FCSAPM) were employed as paraspinal muscle metrics.

Our results indicate that CT Hu and PVMQ serve as significant independent predictors across all evaluated segments. The distinct predictive performances among the bone and muscle parameters can be largely attributed to their inherent measurement methodologies and differing biomechanical roles (26). For bone evaluation, the stronger performance of CT Hu compared with BMD and VBQ may be explained primarily by its local spatial specificity. Although BMD and CT Hu are both related to bone mineral content, DXA-derived BMD represents an areal and relatively global measurement, commonly obtained from the lumbar spine or hip, and may not accurately reflect the local biomechanical environment adjacent to an individual fractured vertebra (27). In addition, lumbar BMD can be influenced by degenerative changes, osteophytes, vascular calcification, and other extra-osseous factors, which may weaken its ability to capture focal vertebral fragility (28). Compared with VBQ, CT Hu may also provide more direct segment-specific information. CT Hu was quantified by extracting the trabecular attenuation of the vertebrae immediately adjacent to the fractured segment. This localized approach directly reflects the cancellous bone condition and load-bearing capacity surrounding the injury site. In contrast, the VBQ score is a more global MRI-derived index calculated across the L1–L4 vertebrae, reflecting overall lumbar vertebral bone quality rather than the local biomechanical status adjacent to the fractured vertebra (29). Since vertebral compression is primarily a focal structural failure under axial loading, adjacent-segment CT Hu may provide a more direct reflection of segment-specific collapse risk than either global areal BMD or pan-lumbar VBQ. Conversely, for muscle evaluation, the PVMQ score demonstrated a more pronounced and consistent contribution compared to the localized FIR. In this study, FIR was assessed specifically at the levels adjacent to the fracture, mirroring the highly localized approach of CT Hu. However, the PVMQ score evaluates the mean signal intensity ratio across multiple lumbar levels (L1 to L4), reflecting the comprehensive quality of the entire lumbar paraspinal musculature. Biomechanically, while a vertebral fracture is a localized event, the paraspinal muscles act synergistically as an integrated, multi-segmental “bowstring” system to maintain spinal sagittal balance (30). Therefore, the overall degenerative state of the paravertebral muscle (captured by PVMQ) exerts a more profound and dominant impact on dynamic spinal stability than the localized fat infiltration degree (FIR) at isolated cross-sections.

Consistent across all segments, CT Hu accounted for the largest proportion of the explained variance in SACR-FVB (ranging from 52.7% to 68.2%). This validates that the localized microarchitectural integrity of the cancellous bone remains the primary mechanical scaffold resisting axial compression irrespective of the specific segmental level. Furthermore, variance decomposition revealed that the combined contribution of paraspinal muscle parameters (PVMQ and FIR) to the explained variance reached its maximum at the L1 segment (43.7%). From a biomechanical and anatomical perspective, L1, as the first lumbar segment completely devoid of thoracic cage protection, exhibits significantly increased mobility and directly bears the concentrated stress from the upper trunk (30). Having lost the static support provided by the thoracic cage, the L1 segment relies heavily on the dynamic compensation of the paraspinal musculature to maintain sagittal balance and buffer downward axial loads; therefore, the role of these muscles in preserving the structural stability of this segment becomes exceptionally critical.

RCS modeling was used to evaluate the continuous dose-response associations between the retained predictors and SACR-FVB. ANOVA tests for non-linearity did not identify significant non-linear deviations across the evaluated segments, suggesting that vertebral biomechanical vulnerability may increase gradually with declining bone quality and progressive paravertebral muscle degeneration. To improve clinical interpretability, we further derived exploratory segment-specific reference values corresponding to a predicted SACR-FVB of 20%, with other covariates fixed at their median values. The exploratory reference values for CT Hu were approximately 99.1, 97.7, and 114.4 at T11, T12, and L1, respectively, whereas the corresponding PVMQ values were 0.323, 0.277, and 0.201. These findings suggest that segment-specific CT Hu and PVMQ reference values may help clinicians interpret the risk of clinically meaningful vertebral collapse during quantitative monitoring.

The significance of this study lies in establishing a highly segment-specific biomechanical framework for the thoracolumbar spine. Our findings demonstrate that local bone quality and overall muscle quality better reflect the true compressive strength of the spine, providing crucial evidence for tailoring individualized preventive and rehabilitative strategies.

Regarding clinical intervention, for lower segments lacking thoracic protection (e.g., L1), the core intervention must include aggressive rehabilitation of the overall paraspinal musculature to reconstruct the posterior tension band. Regarding disease monitoring, the continuous risk gradients revealed by RCS highlight the limitations of traditional paradigms that rely on single rigid cut-offs (e.g., BMD T-scores). Utilizing local bone attenuation and overall muscle quality as continuous quantitative monitoring tools during opportunistic screening enables the early capture of progressive subclinical deterioration, thereby successfully identifying high-risk populations and securing a crucial time window for intervention before vertebral fractures occur. Furthermore, The choice between conservative and interventional management is relevant to the interpretation of biomechanical risk factors. Tabanli et al. (31) reported that patients undergoing vertebroplasty/kyphoplasty had a lower incidence of new fractures than those treated conservatively, suggesting that restoring vertebral height and stabilizing the motion segment may alter local load-sharing. This aligns with our biomechanical framework, and segment-specific variations in bone and muscle quality may also affect adjacent-level fracture risk after intervention. Future studies incorporating pre-treatment imaging with post-treatment outcomes are warranted to further clarify these interactions.

Although MRI-derived parameters such as VBQ, PVMQ and FIR offer significant predictive value, their clinical applicability in routine practice at non-specialized centers warrants objective consideration. The manual delineation of muscular cross-sectional areas and the extraction of precise signal intensities require specific imaging processing software and advanced radiological expertise. These technical requirements may currently limit the extensive feasibility and cross-institutional reproducibility of such measurements. The future integration of automated segmentation algorithms and deep learning models is necessary to standardize these multidimensional imaging metrics and enhance their utility across diverse healthcare settings.

Several limitations of the present study must be acknowledged. First, the retrospective and cross-sectional design inherently precludes the establishment of definitive causal relationships and limits the control of treatment-related confounders. Variations in anti-osteoporotic medication regimens, physical rehabilitation status, analgesic use, and baseline physical activity levels among patients may independently influence musculoskeletal quality and bone metabolism, thereby affecting the severity of vertebral collapse. These variables were not fully adjusted for in the current multivariate models. Second, as a single-center study, the inherent selection bias and localized demographic profile imply that the generalizability of our findings across broader populations remains to be externally verified. Third, the study cohort was exclusively restricted to female patients. Given the well-documented gender disparities in hormone-driven bone metabolism and age-related paraspinal muscle mass distribution, these segment-specific load-sharing paradigms cannot be directly extrapolated to male populations. Finally, the reliance on static, supine-positioned imaging acquisitions fails to capture the real-time kinematic forces and shear stresses acting on the spine during upright weight-bearing activities. Future prospective, multicenter studies incorporating both genders and dynamic biomechanical testing are warranted to validate and refine these findings.

## Conclusion

This study highlights the significant role of bone and muscle quality in predicting vertebral collapse in OVCFs. CT Hu and PVMQ were identified as key independent predictors of vertebral collapse severity. Our findings suggest that localized bone quality remains the primary structural support against axial compression, but as we move down the segments, the role of muscle becomes increasingly important. Continuous monitoring of bone and muscle quality could help identify high-risk patients early, allowing for timely interventions.

In conclusion, this research emphasizes the importance of a multidimensional approach to assessing spinal health, offering valuable insights for personalized fracture risk evaluation and intervention strategies.

## Data Availability

The raw data supporting the conclusions of this article will be made available by the authors, without undue reservation.
