# Supplementary material for: Segment-specific prediction of vertebral collapse severity in osteoporotic fractures
Source: Front Endocrinol (Lausanne). 2026 Aug 7;17:1867204. doi: 10.3389/fendo.2026.1867204 (PMC13493297; doi:10.3389/fendo.2026.1867204)
Supplement: Supplementary file 1 [file Table1.docx]

**Sensitivity analyses using conventional forced-entry multivariable regression**

To assess whether the main findings were dependent on LASSO-based variable selection, conventional forced-entry multivariable linear regression was performed for each fractured segment. SACR-FVB was used as the dependent variable, and clinically predefined variables including age, BMI, BMD, CT Hu, VBQ, PVMQ, and FIR were simultaneously entered as independent variables. Multicollinearity was assessed using variance inflation factors, and LMG relative importance analysis was repeated in the forced-entry models.

**Supplementary Table S1. Sensitivity analysis using conventional forced-entry multivariable linear regression.**

| **Segment** | **Variable** | **B** | **SE** | **Standardized *β*** | ***t* value** | ***P* value** |
| --- | --- | --- | --- | --- | --- | --- |
| **T11** | Age | -0.000426 | 0.000728 | -0.044 | -0.584 | 0.561 |
|  | BMI | -0.001428 | 0.001709 | -0.063 | -0.836 | 0.406 |
|  | BMD | 0.004632 | 0.010400 | 0.041 | 0.445 | 0.657 |
|  | CT Hu | -0.002319 | 0.000444 | -0.627 | -5.225 | **＜0.001** |
|  | VBQ | -0.000841 | 0.008869 | -0.011 | -0.095 | 0.925 |
|  | PVMQ | 0.215360 | 0.091998 | 0.263 | 2.341 | **0.022** |
|  | FIR | 0.028554 | 0.077598 | 0.036 | 0.368 | 0.714 |
|  | Age | -0.000327 | 0.000738 | -0.035 | -0.443 | 0.659 |
| **T12** | BMI | -0.000918 | 0.001780 | -0.042 | -0.515 | 0.608 |
|  | BMD | -0.000021 | 0.010550 | -0.0002 | -0.002 | 0.998 |
|  | CT Hu | -0.001754 | 0.000384 | -0.493 | -4.566 | **＜0.001** |
|  | VBQ | 0.008724 | 0.005371 | 0.153 | 1.624 | 0.109 |
|  | PVMQ | 0.137000 | 0.063500 | 0.229 | 2.157 | **0.035** |
|  | FIR | 0.062070 | 0.057430 | 0.107 | 1.081 | 0.284 |
| **L1** | Age | -0.000105 | 0.001890 | -0.005 | -0.056 | 0.956 |
|  | BMI | 0.002493 | 0.003133 | 0.065 | 0.796 | 0.429 |
|  | BMD | -0.008335 | 0.014720 | -0.057 | -0.566 | 0.573 |
|  | CT Hu | -0.002119 | 0.000491 | -0.498 | -4.316 | **＜0.001** |
|  | VBQ | 0.006398 | 0.008478 | 0.065 | 0.755 | 0.454 |
|  | PVMQ | 0.226431 | 0.090406 | 0.274 | 2.505 | **0.015** |
|  | FIR | 0.096212 | 0.084351 | 0.112 | 1.141 | 0.259 |

Model performance: T11: R-squared = 0.607, adjusted R-squared = 0.571, *P* < 0.001; T12: R-squared = 0.630, adjusted R-squared = 0.589, *P* < 0.001; L1: R-squared = 0.640, adjusted R-squared = 0.596, *P* < 0.001. B, unstandardized regression coefficient; SE, standard error; Significant values are highlighted in bold.

**Supplementary Table S2. Multicollinearity assessment using VIF.**

| **Segment** | **Age** | **BMI** | **BMD** | **CT Hu** | **VBQ** | **PVMQ** | **FIR** |
| --- | --- | --- | --- | --- | --- | --- | --- |
| T11 | 1.120 | 1.105 | 1.655 | 2.819 | 2.450 | 2.472 | 1.884 |
| T12 | 1.100 | 1.165 | 1.605 | 2.012 | 1.525 | 1.966 | 1.698 |
| L1 | 1.069 | 1.068 | 1.648 | 2.138 | 1.200 | 1.949 | 1.541 |

All VIF values were below 3, indicating no substantial multicollinearity in the forced-entry models.

**Supplementary Table S3. LMG variance decomposition in the forced-entry sensitivity models.**

| **Segment** | **Age** | **BMI** | **BMD** | **CT Hu** | **VBQ** | **PVMQ** | **FIR** |
| --- | --- | --- | --- | --- | --- | --- | --- |
| T11 | 1.18% | 3.18% | 6.85% | 44.61% | 15.10% | 17.96% | 11.12% |
| T12 | 0.75% | 2.56% | 8.65% | 41.23% | 16.19% | 21.04% | 9.59% |
| L1 | 0.65% | 0.57% | 10.89% | 44.01% | 5.04% | 28.55% | 10.29% |

Values indicate the proportional contribution of each clinically predefined predictor to the explained variance in SACR-FVB. In all three segments, CT Hu remained the largest contributor to the explained variance.

**Supplementary Table S4. Exploratory RCS-derived reference values.**

| **Segment** | **Variable** | **Approximate value at**  **predicted SACR-FVB = 20%** |
| --- | --- | --- |
| T11 | CT Hu | 99.1 |
| T11 | PVMQ | 0.323 |
| T12 | CT Hu | 97.7 |
| T12 | PVMQ | 0.277 |
| L1 | CT Hu | 114.4 |
| L1 | PVMQ | 0.201 |

The reference values indicate the approximate CT Hu or PVMQ values at which the fitted predicted SACR-FVB reached 20%. These values were derived from fitted RCS curves and should be interpreted as exploratory risk-reference values rather than validated clinical thresholds.
